# Supplementary material for: The genetic basis and fitness consequences of sperm midpiece size in deer mice
Source: Nat Commun. 2016 Dec 2;7:13652. doi: 10.1038/ncomms13652 (PMC5146288; doi:10.1038/ncomms13652)
Supplement: Supplementary Information — Supplementary Figures 1-4, Supplementary Tables 1-2, Supplementary Note 1 and Supplementary References. [file ncomms13652-s1.pdf]

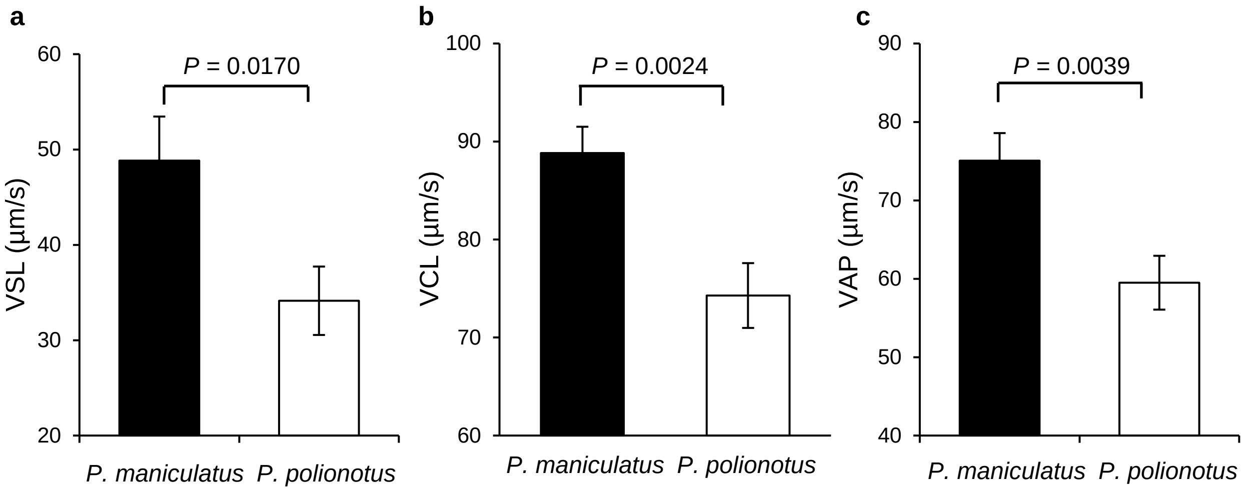

Supplementary Figure 1. ***Peromyscus* sperm velocity**. Mean±SE sperm velocity measured in *P. maniculatus* and *P. polionotus* males in three ways (n=9 individual; n=76-549 sperm/individual; t-test): (**a**) straight-line velocity (VSL), (**b**) curvilinear velocity (VCL), and (**c**) average path velocity (VAP). All three measures show that *P. maniculatus* sperm cells are significantly faster than *P. polionotus* cells. Note truncated y-axis.

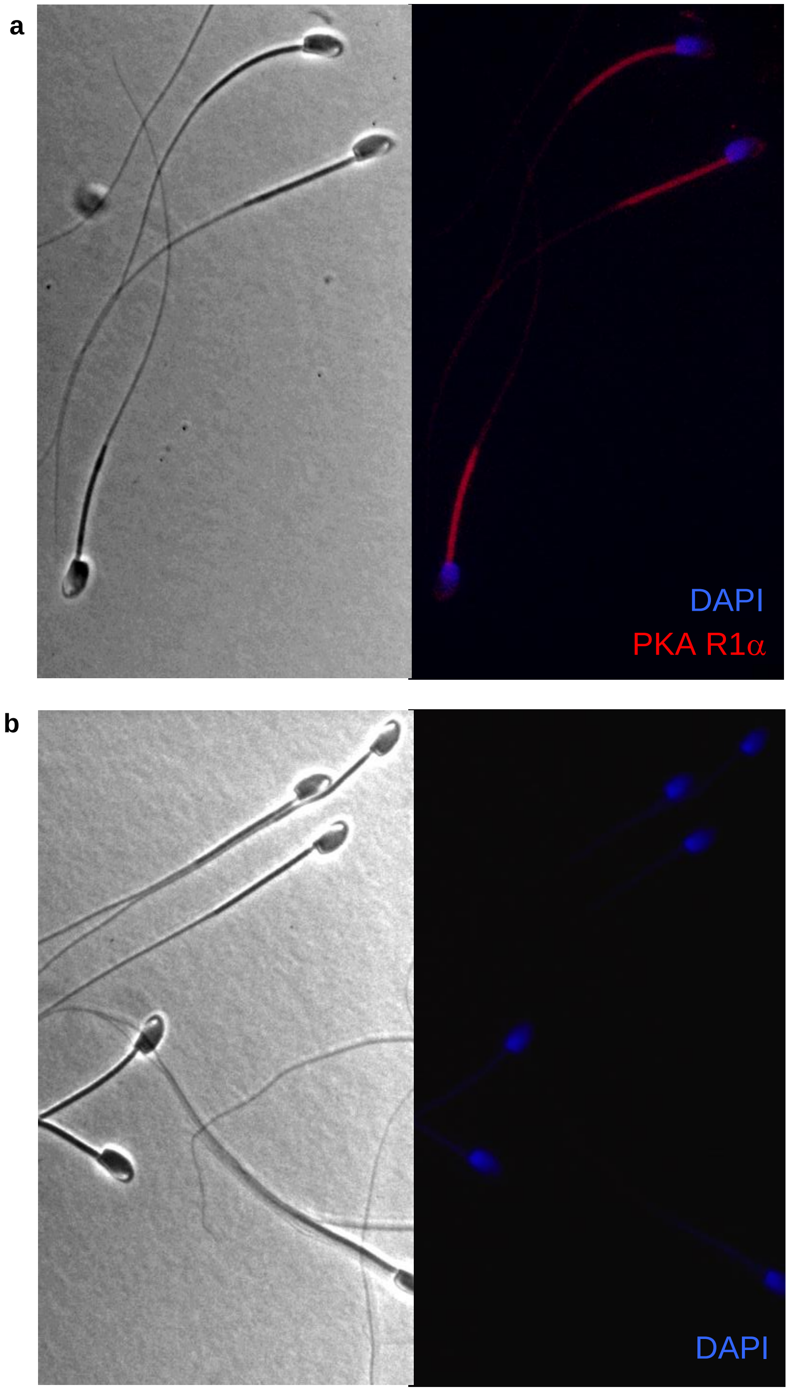

Supplementary Figure 2. PKA R1α localization in *Peromyscus* sperm cells. Phase-contrast images of epididymal sperm cells from *P. maniculatus* at 1000X magnification (left) and the same cells stained for DAPI to visualize the nuclei and with (**a**) or without (**b**) anti-PKA R1α primary antibody (right). Both treatment (**a**) and control (**b**) cells were subjected to identical manipulation including exposure to the secondary antibody, Alexa Fluor 546. The figure shows that the PKA R1α protein is primarily located in the midpiece.

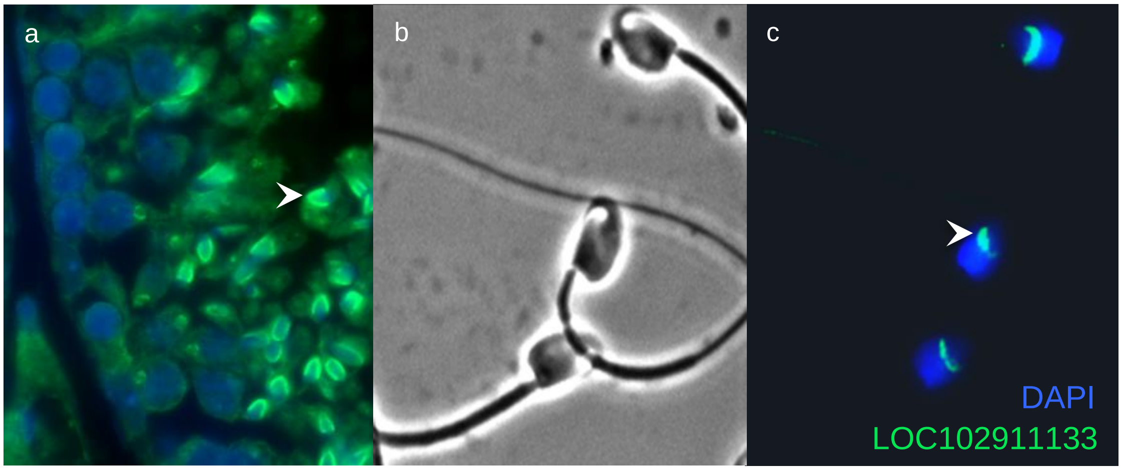

Supplementary Figure 3. LOC102911133 Protein localization in *Peromyscus* testis and sperm cells. Immunofluorescence of anti-LOC102911133 and Alexa Fluor 488 (green) and DAPI (blue) in (**a**) cross-section *P. maniculatus* testes, (**b**) phase-contrast image of epididymal sperm cells, and (**c**) and their immunofluorescence profile. LOC102911133 (arrow heads) localizes to the equatorial segment on sperm heads in both testis sections (a) and mature sperm (c).

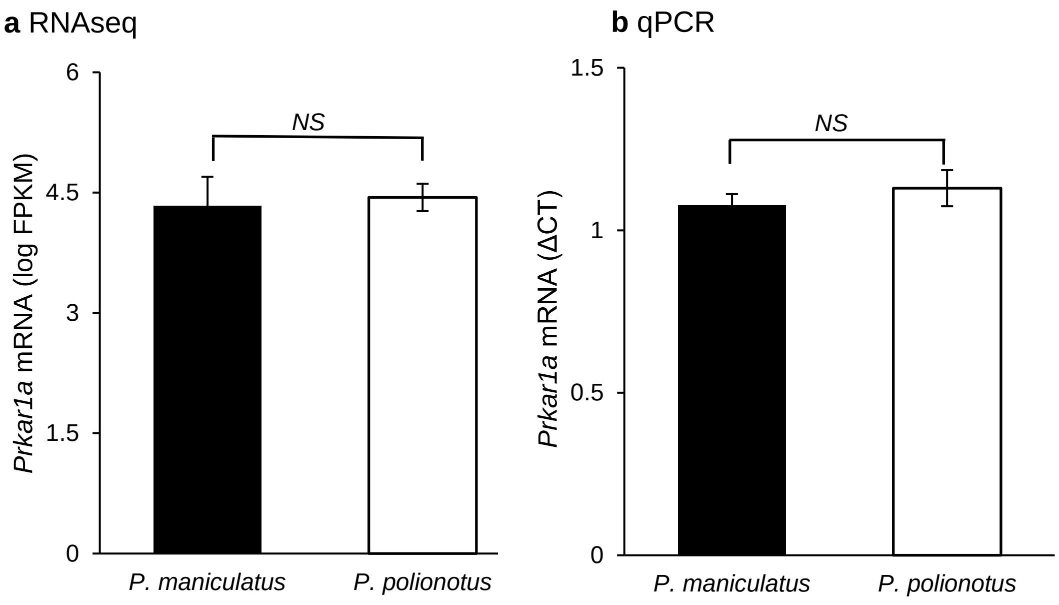

Supplementary Figure 4. *Prkar1a* mRNA expression. Mean±SE of *Prkar1a* mRNA in whole testis tissue quantified by (**a**) RNAseq (*n*=8) and (**b**) qPCR (*n*=4). T-test; *NS* = p-value > 0.05.

Supplementary Table 1. Fine-scale mapping assay, primers and probes for 10 loci.

Locus	Assay*	Primers (5'-3')	Probe (5'-3')
Foxj1	TaqMan	GCTCCACTCAGCCTTGTATTCT<br>CTGCTCCTCAAAGTCATTCATGTCT	CTTCCTTGC(G/A)TTCTTAG
Kcnj16	TaqMan	CGATGGCCCCAGAGAATTTCC<br>CTGGGACATCCCACCAGTC	TAGAAGTTCCTA(C/T)GTCCCC
Amz2†	sequence	ATGGCTCAAGGGCTACTGTG<br>AAGAACGCCAGGATGTGC	-
Prkar1a	TaqMan	CCTGCCAACATTGAGTTAGGTTTT<br>TGGTCATACAGTATTGTTCGTTTCACA	ATAGGCC(T/C)CAGGCATT
Abca8b†	sequence	TTGTTGGCATGTCTGTGAGGCA<br>ACTGGCTTGCCCTGGTTTGCT	-
Prkca	TaqMan	CCCATCCCTGCACAGACA<br>ACAAATCCTGGTAAACCAAGTCACT	CCTGT(G/C)AAACAGAAGC
Fmnl1	TaqMan	TATTCTGTCCAATGGTGTTCTGTCA<br>TGGGCTCAACACTGTC	AGAGCTAAGGTCGTT(G/A)GT
Slc25a39	TaqMan	GTGGACTGCTCAGTGTAGGAA<br>GTTACGGTTATTGTTTTGATTCTCAGCAT	CCTGAA(T/C)GAATCCC
Klhl10	TaqMan	GGTCAAGTTTAGTTATTCTTTGCAAATGCT<br>CTCTCATTAAGCCCTGGGATCTG	AGGGACTGT(C/-)CCCAAGAG
Ttll6	Enzyme<br>Kpn1	GGTTTACGTGCTGGTGACCT<br>GCTGCCAGAATGAGCATCTT	-

***Assay indicates whether SNP genotype was assessed using a TaqMan® SNP Genotyping Assay, Sanger sequencing of the DNA fragment, or with an informative restriction enzyme.

† Only F_2 hybrids that showed evidence of a recombination event between *Foxj1* and *Fmnl1* (*n*=13) were genotyped at *Amz2* and *Abca8b*.

Supplementary Table 2. Annotated genes residing in 1cM genomic region associated sperm midpiece length.

Gene symbol	Gene	Position*	Description of gene function	Human disease phenotype(s)	Sperm references
Slc16a6	Solute carrier family 16, member 6	11: 109,450,855-109,473,596	Catalyzes rapid cross-membrane transport of monocarboxylates[16]	-	-
Arsg	Arylsulfatase G	11: 109,450,845-109,573,330	Catalyzes hydrolysis of sulfate esters[17]	hypertension (high blood pressure)[17]	-
Wipi1	WD repeat domain, phosphoinositide interacting 1	11: 109,573,521-109,611,389	Degrades cytosolic components (autophagy)[18]	-	-
Prkar1a	Protein kinase, cAMP-dependent, regulatory, type I, alpha	11: 109,649,395-109,669,659	Regulates metabolism, proliferation, differentiation, and apoptosis[19]	Carney Complex (cancer of connective tissue) and associated male infertility[20,21]	1-14
Fam20a	Family with sequence similarity 20, member A	11: 109,669,492-109,722,510	Involved in biomineralization of enamel and tooth eruption[22]	hypoplastic amelogenesis imperfecta (dental enamel defects)[22,23]	-

* Position in base pairs in the *Mus musculus* GRCm38 reference genome sequence[15].

† Functional and transcriptional literature review of all known genes found in the region was conducted to identify those associated with male fertility, spermatogenesis, sperm motility, and/or specifically or more highly expressed in the testes or during spermatogenesis. Review preformed in PubMed (using each of those terms as search items, as well as “sperm”, gene name and symbol) and several online databases: UCSC Genome Browser[16], UniProt[17], Mouse Genome Database[15], and Rat Genome Database[18].

Supplementary Note 1

Fitness assays

Male fitness has multiple components; we therefore chose to assess how sperm morphology independently affects the two components of fitness at the focus of our study – sperm competitive ability and male reproductive success.

First, we directly tested the contribution of midpiece length to sperm competitive ability using swim-up assays, a standard fertility technique employed to determine which sperm in a sample are most motile. Specially, we conducted three independent assays to compete variable sperm in different contexts: (1) competitions between sperm of different species (*P. maniculatus* and *P. polionotus*), which maximize the difference in sperm morphology; (2) competitions between males of the same promiscuous species (*P. maniculatus*), which are the most biologically relevant scenario; and (3) competitions among sperm from a single male, which are the best controlled experiments as the sperm are most similar in other morphological and physiological aspects.

Second, we measured male reproductive success in a single-male, single-female context, which importantly is the only natural mating assay that avoids inherent biases resulting from pre-mating female choice, male competition, and cryptic female choice. When we compared midpiece and flagellum lengths of the F_2 males that did and did not sire offspring using two-tailed unpaired t-tests to determine if sperm morphology influences reproductive success: we found evidence for an affect of midpiece (t-test: $P_{midpiece}$=0.041, df=84, Fig. 8) but not flagellum length (t-test: $P_{flagellum}$=0.39, df=84). Attempts at competitive mating assays (two males housed with one or two females) revealed that male aggression in these species could be extreme and inhibit multiple mating by females (and thus sperm competition). We also were unable to achieve reliable results from artificial insemination and *in vitro* fertilization in these (non-model) species; yet as methods designed to induce pregnancy when natural mating fails,

reproductive success rates from such assays, which intentionally bypass many of the barriers to fertilization that sperm would naturally encounter, produce false positives (by design) and therefore can lead to result that are difficult to interpret. Moreover, sequential matings with multiple males are likely to produce order effects.

Analysis of genes located in QTL peak

Using a genetic breakpoint analysis, we confirmed support for *Prkar1a* by narrowing the 3.3cM interval to 1cM (Fig. 5). This region contains only four other annotated protein-coding genes (*Slc16a6, Arsg, Wipi1,* and *Fam20a*), which are unlikely candidates for sperm midpiece length on the basis of their known function (Supplementary Table 1). *Prkar1a* has been repeatedly assigned a causal role in sperm morphology, motility and in determining fertility in laboratory mice and humans[1-14]. By contrast, none of the other four genes in the QTL interval have been associated with these reproductive functions (Supplementary Table 1).

In addition to the known genes within our QTL interval, we also explored a predicted gene located ~150kb from *Prkar1a*, *LOC102911133* (a homolog to *1700012B07Rik* in *Mus musculus)*. Although very little has been reported about this predicted gene, and its structure and properties give few clues to its function, it is highly and specifically expressed in the testis and germ cells of male rodents[15]. To understand which cellular structure(s) are associated with this predicted gene, we designed a custom antibody for the predicted protein and localized it in testes sections and mature sperm cells.

To localize the expression of the protein product of the predicted gene *LOC102911133*, we fixed epididymal sperm in 2% paraformaldehyde and 1.25% glutaraldehyde on a microscope slide for 15 min. We then washed the cells in phosphate-buffered saline with 0.1% Tween 20 (PBT) for 15 min, and blocked in PBT with 3% bovine serum albumin (BSA) for 1hr at room temperature (RT). Next we incubated the cells overnight at 4°C with using one of two custom primary antibodies prepared by

GenScript (Piscataway, NJ) with the following amino acid sequences, Peptide1: IPHEKKKENKNHEDC and Peptide2: CLSLGKDDSHHEKEK diluted at 1:500 in PBT with 3% BSA. The following day we washed cells in PBT for 1hr at RT 3 times, then incubated with the secondary antibody, Alexa Fluor 488 (Thermo-Fisher #A11029, Waltham, MA), diluted at 1:1000 for 1hr at RT. Cells were then washed in PBT for 1hr at RT 3 times, stained with DAPI (Thermo-Fisher, Waltham, MA) to visualize DNA within cells for 15min at RT, washed a final time in PBT for 15min, and mounted in Fluoromount-G (Southern Biotech, Birmingham, AL). In addition, we controlled for non-specific binding of the secondary antibody by performing a side by side comparison with cells processed identically to the above methods except that instead of treating with the primary antibody, cells were treated solely with PBT with 3% BSA, the secondary antibody and DAPI. We viewed cells at 400X and 1000X magnification on an upright microscope (AxioImager.A1, Zeiss, Jena, Germany). We followed the same procedure for localizing LOC102911133 on 4% paraformaldehyde fixed and paraffin embedded sections of testis tissue. The protein product of LOC102911133 appears to localize to the head of developing and mature sperm cells, and specifically to the equatorial segment in the head of mature cells in *Peromyscus* (Supplementary Fig. 3), unlike PKA R1α, which localizes to the midpiece (Fig. 6). Therefore, based on its spatial expression, LOC102911133 was not a strong candidate for variation in sperm midpiece length.

Supplementary References

1. Burton, K. A. & McKnight, G. S. PKA, germ cells, and fertility. *Physiology* **22,** 40–46 (2007).
2. Burton, K. A. *et al.* Haploinsufficiency at the Protein Kinase A RIα gene locus leads to fertility defects in male mice and men. *Mol. Endocrin.* **20,** 2504–2513 (2006).
3. Reinton, N. *et al.* Localization of a novel Human A-Kinase-Anchoring protein, hAKAP220, during spermatogenesis. *Develop. Biol.* **223,** 194–204 (2000).
4. Miki, K. Identification of tethering domains for Protein Kinase A Type I alpha Regulatory subunits on Sperm Fibrous Sheath Protein FSC1. *J. Biol. Chem.* **273,** 34384–34390 (1998).
5. Vijayaraghavan, S., Goueli, S. A., Davey, M. P. & Carr, D. W. Protein kinase A-anchoring inhibitor peptides arrest mammalian sperm motility. *J. Biol. Chem.* **272,** 4747–4752 (1997).
6. Amieux, P. S. & McKnight, G. S. The essential role of RI alpha in the maintenance of regulated PKA activity. *Ann. N. Y. Acad. Sci.* **968,** 75–95 (2002).
7. Veugelers, M. *et al.* Comparative PRKAR1A genotype-phenotype analyses in humans with Carney Complex and *Prkar1a* haploinsufficient mice. *Proc. Nat. Acad. Sci. U.S.A.* **101,** 14222–14227 (2004).
8. De Mateo, S. *et al.* Marked correlations in protein expression identified by proteomic analysis of human spermatozoa. *Proteomics* **7,** 4264–4277 (2007).
9. Wieacker, P. *et al.* Male infertility as a component of Carney complex. *Andrologia* **39,** 196–197 (2007).
10. Hermo, L., Pelletier, R. M., Cyr, D. G. & Smith, C. E. Surfing the wave, cycle, life history, and genes/proteins expressed by testicular germ cells. Part 3: Developmental changes in spermatid flagellum and cytoplasmic droplet and interaction of sperm with the zona pellucida and egg plasma membrane. *Microsc. Res. Tech.* 320-363 (2009).
11. Hermo, L., Pelletier, R. M., Cyr, D. G. & Smith, C. E. Surfing the wave, cycle, life history, and genes/proteins expressed by testicular germ cells. Part 4: Intercellular bridges, mitochondria,

nuclear envelope, apoptosis, ubiquitination, membrane/voltage-gated channels, methylation/acetylation, and transcription factors. *Microsc. Res. Tech.* **73,** 364–408 (2010).

12. Byrne, K., Leahy, T., McCulloch, R., Colgrave, M. L. & Holland, M. K. Comprehensive mapping of the bull sperm surface proteome. *Proteomics* **12,** 3559–3579 (2012).
13. Hwang, K. *et al.* Mendelian genetics of male infertility. *Ann. N. Y. Acad. Sci.* **1214,** E1–E17 (2011).
14. Willis, B. S., Niswender, C. M., Su, T., Amieux, P. S. & McKnight, G. S. Cell-type specific expression of a dominant negative PKA mutation in mice. *PLoS ONE* **6,** e18772 (2011).
15. Blake, J. A. *et al.* The Mouse Genome Database: integration of and access to knowledge about the laboratory mouse. *Nucleic Acids Res.* **42,** D810–817 (2014).
16. Karolchik, D. *et al.* The UCSC Genome Browser database: 2014 update. *Nucleic Acids Res.* (2014).
17. Consortium, T. U. The Universal Protein Resource (UniProt) in 2010. *Nucleic Acids Res.* (2010).
18. Shimoyama, M. *et al.* The Rat Genome Database 2015: genomic, phenotypic and environmental variations and disease. *Nucleic Acids Res.* **43,** D743–750 (2015).
