# Supplementary material for: The genetic basis and fitness consequences of sperm midpiece size in deer mice
Source: Nat Commun. 2016 Dec 2;7:13652. doi: 10.1038/ncomms13652 (PMC5146288; doi:10.1038/ncomms13652)
Supplement: Peer Review File [file ncomms13652-s2.pdf]

PEER REVIEW FILE

Transferred manuscripts:

Editorial Note: this manuscript has been previously reviewed at another journal that is not operating a transparent peer review scheme. This document only contains reviewer comments and rebuttal letters for versions considered at Nature Communications.
